# The relationship between smoking and recurrent aphthous stomatitis: A Mendelian randomization study

**DOI:** 10.18332/tid/199253

**Published:** 2025-01-15

**Authors:** Yujiao Hu, Cheng Chen, Fei Yu, Jin Zhang, Hui Zeng

**Affiliations:** 1 Department of Stomatology, Xi’an Jiaotong University Stomatology Hospital, Xi’an, China; 2 Department of Stomatology, Nanchang University, Nanchang, China; 3 School of Stomatology, Xi’an Medical University, Xi’an, China

**Keywords:** smoking, recurrent aphthous stomatitis, mouth ulcers, mendelian randomization

## Abstract

**INTRODUCTION:**

Existing research suggests an association between smoking and the incidence of recurrent aphthous stomatitis (RAS); however, the causal relationship remains ambiguous. We employed Mendelian randomization (MR) to clarify the potential causal association between smoking and the risk of developing RAS.

**METHODS:**

We utilized genome-wide association study (GWAS) sequencing data related to smoking from the Finnish database as instrumental variables (IVs) and GWAS data for RAS from the UK Biobank (UKB) as the outcome to perform a two-sample MR analysis. The selection of IVs was rigorously controlled according to the three principal assumptions of relevance, independence, and exclusivity. The primary analytical methods utilized were inverse variance weighting (IVW) and weighted median (WM), supplemented by MR-Egger, simple mode, and weighted mode techniques to infer causality between smoking and RAS. Sensitivity analyses were conducted using MR-PRESSO, Cochran's Q, and the MR-Egger intercept to ensure the robustness of the findings.

**RESULTS:**

The findings from the IVW and WM analyses suggest a causal association between smoking and an elevated risk of RAS (IVW: OR=1.003; 95% CI: 1.0002–1.005, p=0.033; WM: OR=1.003; 95% CI: 1.00006–1.007, p=0.044). Compared to non-smokers, smokers have a 0.3% increase in the risk of RAS. Furthermore, the sensitivity analysis did not reveal any inconsistencies that would contradict the MR results.

**CONCLUSIONS:**

Our findings provide preliminary evidence of a potential causal relationship between smoking and the risk of RAS, which may contribute to a deeper understanding of the underlying mechanisms. Further research is needed to confirm these results and explore their implications for clinical practice.

## INTRODUCTION

Recurrent aphthous stomatitis (RAS) represents the most prevalent condition affecting the oral mucosa, with its prevalence estimated to range from 5% to 25% within the general population^1^. Recurrent aphthous stomatitis (RAS) represents the most prevalent condition affecting the oral cavity. These ulcers present as ulcerative lesions on any soft tissue within the mouth and are commonly associated with sensations of pain, burning, and general discomfort at rest, which are exacerbated by oral activities such as speaking and chewing^2^. Although the majority of RAS are self-limiting, their recurrent nature and the intensity of the associated pain can substantially diminish the well-being of individuals affected by the condition^3^. An explanation for the etiology of RAS is multifaceted, potentially involving genetic predispositions, local trauma, immune system dysfunctions, nutritional deficiencies, infections, and psychological stress^4^. The pharmacological management of RAS presents significant challenges, with a paucity of efficacious therapeutic agents. Furthermore, the absence of a definitive etiological understanding and well-established treatment protocols, underscores the imperative for continued research into its pathogenesis. Such investigations are crucial for informing public oral health interventions.

Smoking represents a critical lifestyle factor and constitutes a significant public health concern on a global scale. Chronic diseases associated with it include respiratory disorders, cardiovascular disease, and various forms of cancer^5^. Furthermore, smoking may adversely affect oral health, contributing to an elevated risk of oropharyngeal cancer and periodontal disease^6,7^. A study by Chaudhuri et al.^8^ suggested an association between smoking addiction and the incidence of RAS. Conversely, Kudsi et al.^9^ found that smoking was not associated with oral mucosal lesions. Nevertheless, the findings from these observational studies exhibit inconsistencies, and the existence of possible confounding variables, coupled with the inherent risk of reverse causation, hinders the ability to establish a conclusive potential causal link between smoking and RAS.

Mendelian randomization (MR) is a methodological approach that employs naturally occurring genetic variations, specifically single nucleotide polymorphisms (SNPs), associated with a particular exposure as instrumental variables (IVs) to clarify the causal relationship between exposure factors and target outcomes. Given that genetic variations are randomly assigned in accordance with Mendel’s second law, this methodology parallels the random allocation observed in randomized controlled trials. Additionally, the occurrence of these genetic mutations precedes the manifestation of the phenotype; therefore, confounding variables and reverse causality are mitigated. With the swift and extensive advancement of databases for genome-wide association studies (GWAS), MR analysis methods have been widely utilized in epidemiological research. It has been shown that MR analyses provide more reliable and robust results than traditional observational studies^10^.

Smoking may play a causal role in the occurrence of RAS. Taking this into account, our study seeks to further investigate this association by utilizing smoking-related genetic variations as non-confounding instruments and employing MR methods.

## METHODS

### Study design

**Figure 1:**
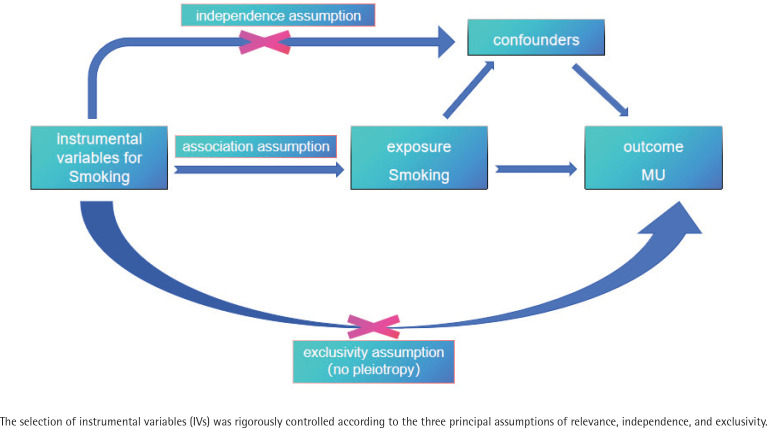
Diagram of MR study design

We utilize two-sample MR to assess the association between smoking and RAS. The core assumptions of two-sample MR analysis encompass three fundamental conditions. First, the association assumption, which states that significant associations exist between the instrumental variable (IV, SNPs related to exposure) and the exposure factor. Second, the independence assumption stipulates that the IVs must be independent of each other. Third, the exclusivity assumption posits that the IVs affect the outcome solely through the exposure factor, without any influence via alternative pathways (i.e. no pleiotropy). A diagram of the MR study design is shown in Figure 1. The validity of these assumptions is essential to ensure that causal effect estimates remain unbiased.

### Data sources

The majority of GWAS data are derived from European cohorts, and our study utilizes publicly accessible data from these populations. To ensure minimal sample overlap in two-sample MR, we selected two independent cohorts: the Finnish database^11^ and the UK Biobank (UKB)^12^. The UKB is a comprehensive biomedical database and research resource encompassing a cohort of 498697 individuals of European descent residing in the United Kingdom, including data on 7221 distinct participant phenotypes. The RAS GWAS dataset is derived from the UKB database. The RAS sample predominantly comprises data on current oral ulcer status and the history of oral ulcers, primarily derived from self-reports and medical records, and contains 60077 samples. The Finnish database comprises genetic and health information from 453733 Finnish participants of European ancestry. The smoking-related samples utilized in our study are derived from this database. The smoking-related genetic variations predominantly encompass data pertaining to active smoking behavior, specifically indicating whether individuals engage in smoking, which is primarily sourced from medical records, questionnaires, and the national health database. The summary statistics of these samples are derived from comprehensive population cohort studies conducted in Finland, encompassing a total of 166614 samples (summary data from all GWAS used in the current study are shown in Supplementary file Table 1). The datasets utilized in our study were meticulously selected according to their relevance to the research objectives and the accessibility of comprehensive data. These datasets have demonstrated the capacity to furnish high-quality information on genetic variations associated with smoking and RAS.

### Extraction of IVs

In our two-sample MR, the selection of IVs (SNPs associated with smoking) is of paramount importance. To satisfy the three fundamental assumptions of two-sample MR and facilitate the robust execution of MR analysis, the SNPs ultimately employed for both MR and sensitivity analyses must be chosen according to the following criteria: Initially, we conducted a screening for SNPs associated with smoking that achieved a genome-wide significance threshold (p<5×10^-6^) to address the first association hypothesis. Given the number of significant loci identified, we subsequently employed a more permissive significance threshold. Additionally, the assumption necessitates that SNPs are independent of one another. To mitigate potential biases arising from linkage disequilibrium (LD) among SNPs, it is imperative that the selected SNPs exhibit minimal association. To achieve this, we employed LD pruning, utilizing an LD coefficient threshold of r^2^<0.001 within a 10000-kilobase window, ensuring that the SNPs reflect distinct genetic signals. At the same time, we calculated the statistical intensity measure F value of the remaining SNPs, where F=R^2^(N-2)/(1-R^2^) and R^2^=2×EAF×MAF×β^2^. Here, EAF represents the effect allele frequency, MAF denotes the minor allele frequency, and β represents the effect size of the estimated effect allele on the exposure, and N is the sample size^13^. It reflects the strength of each IV’s effect on the exposure phenotype. We excluded SNPs with F<10, to avoid bias caused by weak instruments^14^. Third, within the genetic variation data pertaining to RAS (specify what RAS stands for if necessary), it is imperative to identify the previously mentioned SNPs while excluding those SNPs that exhibit a strong association with RAS (p<5×10^-6^). This exclusion is necessary to ensure that the remaining SNPs are influenced solely by smoking as the exposure factor, without the involvement of alternative pathways, thereby satisfying the exclusivity assumption of the hypothesis. Fourth, to facilitate the seamless execution of subsequent MR and sensitivity analyses, it is essential to harmonize the remaining SNPs using the harmonize data function available in the two-sample MR package. This process involves aligning the allele orientations of the SNPs and eliminating palindromic SNPs that exhibit intermediate allele frequencies. In conclusion, the analysis of MR studies and observational studies concerning the RAS suggests that immune factors, gut microbiota, and psychological characteristics – including anxiety and insomnia^15-17^ – may serve as potential confounding factors influencing the selection of SNPs in our research. To eliminate SNPs that exhibit strong correlations with these confounding factors, the LDlink platform^18^ was utilized to identify the associations between SNPs and confounding factors.

### Statistical analysis

The primary methodologies employed for causal inference in our study include the inverse variance weighted (IVW) approach within the framework of random effects models and the weighted median (WM) method. Additionally, the MR-Egger, simple mode, and weighted mode methods are used as supplementary analyses to observe the distribution and direction of the effects of IVs. The IVW method operates under the assumption that the effects of all IVs on the outcome are mediated exclusively through the exposure factor, thereby precluding horizontal pleiotropy. This methodology assigns weights to the effect estimates of each SNP based on the inverse of their respective variances, subsequently integrating them to derive a comprehensive overall effect estimate^19^. The random effects model offers greater flexibility and robustness in estimation compared to the fixed effects model, particularly when addressing the heterogeneity inherent in IVs. The WM method is considered relatively conservative, as it relies on the effect estimates of each IVs, weighted by the precision of these estimates (typically the inverse of their standard error). Provided that less than half of the SNPs are deemed invalid, the WM method can still yield an accurate causal effect estimate. To assess the relative risk of causal effects, odds ratios (OR) and 95% confidence intervals (CI) were computed. In our study, smoking status and the presence of RAS were categorized as binary variables – distinguishing between smokers and non-smokers, as well as the presence and absence of RAS – according to the classification criteria established in the Finnish database and the UKB. The OR was utilized to quantify the relative impact of transitioning from a non-smoker to a smoker on the risk of developing RAS. Horizontal pleiotropy, which occurs when SNPs are associated with multiple phenotypes, may influence the outcome through pathways other than the phenotype of interest, thereby violating the exclusivity assumption in two-sample MR. To assess the robustness of the IVW estimate, our study performed a series of sensitivity analyses, including the evaluation of the MR-Egger intercept. This intercept term is derived from the MR-Egger regression analysis, which estimates the causal effect of the exposure variable on the outcome. The significance of horizontal pleiotropy is determined by comparing the p-value of the intercept to a predetermined significance threshold of 0.05, with p<0.05 indicating the presence of significant pleiotropy. To ascertain the validity of the MR-Egger regression, we evaluated the instrument strength independent of direct effect (InSIDE) assumption within the analysis. A non-significant p-value suggests that the effect of the IVs is not significantly associated with the direct effect of the exposure on the outcome. This finding supports the validity of the InSIDE assumption and justifies the application of MR-Egger regression. Additionally, the MR-PRESSO test incorporates an outlier-corrected approach to adjust for horizontal pleiotropy by identifying outliers. The global test, a statistical method within MR-PRESSO, evaluates overall pleiotropy by assessing the significance of global pleiotropy based on the magnitude of its p-value^20^. Thereby mitigating the potential for masking or exaggerating the true relationship between exposure factors and outcomes^21^. In our study, Cochran’s Q statistic was employed to assess the heterogeneity among IVs. This statistic is derived from the IVW estimate and is computed based on the deviation between the estimated effect of each SNP and the aggregate IVW estimated effect. The Q-value is expected to follow a chi-squared (χ^2^) distribution and serves as a metric for assessing the extent of heterogeneity in the IV effects. The associated p-value is employed to ascertain the statistical significance of this heterogeneity. A p>0.05 indicates no significant heterogeneity, suggesting that the effects of the various IVs can be considered consistent. The results of the analysis were represented using scatter plots, funnel plots, and leave-one-out analysis. The scatter plot was primarily used to examine the distribution and directionality of the effects of the IVs, as well as to assess the fit of the IVW and other supplementary analytical methods. The funnel plot served to evaluate the heterogeneity of the effects of the IVs. Meanwhile, the leave-one-out analysis was utilized to eliminate the potential influence of causal relationships attributable to the predominant effect of specific SNPs, thereby ensuring the robustness of the results. All statistical analyses in our study were conducted using two-tailed tests, as no specific directional hypothesis was proposed concerning the association between smoking and RAS. All MR analyses conducted in our study utilized the TwoSampleMR package within RStudio version 4.4.1, with p<0.05 deemed statistically significant.

## RESULTS

Smoking-related genetic variations encompass 21288424 SNPs. Following the application of an association threshold (p<5×10^-6^), 635 SNPs were selected. Subsequent application of independence criteria further refined this number to 25 SNPs, each exhibiting an F>10 with values ranging from 594 to 2145. When reviewing the outcome phenotype RAS data, one unmatched SNP (rs112206797) was removed. During the harmonization process, one SNP (rs10820003) was excluded due to its palindromic nature and moderate effect allele frequency. Utilizing the LDlink platform to assess linkage disequilibrium data for each SNP, we retained phenotypes exhibiting genetic effects with an LD r^2^>0.8 (comprehensive data regarding the genetic impact of each SNP-associated phenotype are provided in Supplementary file Table 2). The screening process did not identify any SNPs associated with established confounding factors related to RAS. Consequently, 23 SNPs were selected to serve as IVs for our subsequent MR and sensitivity analyses (comprehensive information regarding each SNP can be found in Supplementary file Table 3).

**Figure 2:**
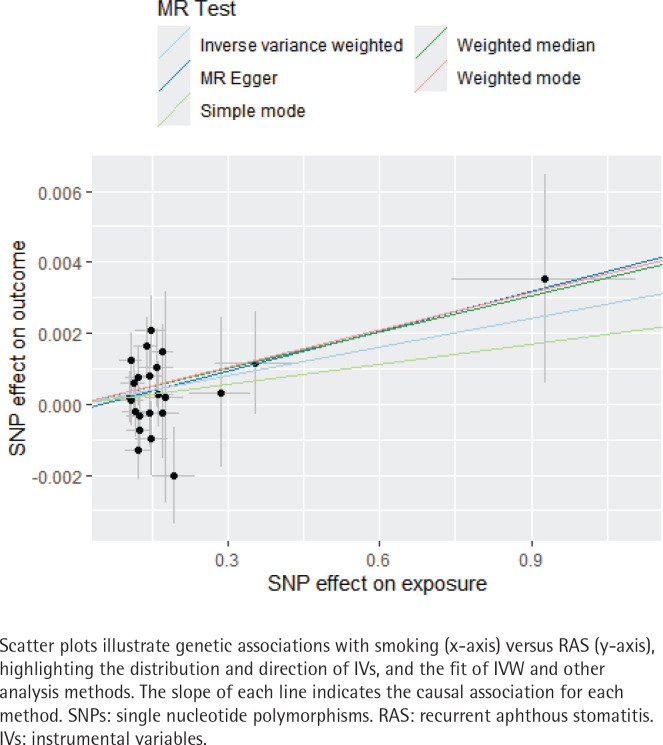
Scatter plot of SNPs effects on exposure and outcome in two-sample MR

Employing IVW and WM methodologies as the primary analytical approaches, the findings indicate a slight positive causal relationship between smoking and an increased risk of RAS (IVW: OR=1.003; 95% CI: 1.0002–1.005, p=0.033; WM: OR=1.003; 95% CI: 1.00006–1.007, p=0.044). Table 1 presents the primary analytical data for the two MR methods, namely IVW and WM. Compared to non-smokers, smokers have a 0.3% increase in RAS risk. The OR values derived from MR-Egger, simple mode, and weighted mode analyses corroborate the trend observed in the IVW results. Figure 2 presents a scatter plot illustrating the consistency and fit of various analytical methods. Despite the small effect size, the statistical significance of the findings supports a potential causal relationship between smoking and the incidence of RAS, indicating that smoking may elevate the risk of developing RAS.

**Table 1:** The main MR analysis results investigating the causal relationship between smoking and recurrent aphthous stomatitis (SNPs=23)

*Exposure phenotype*	*Outcome phenotype*	*Results of MR analysis*					
		*Method*	*p*	*b*	*SE*	*OR*	*95% CI*
Smoking	Recurrent aphthous stomatitis (RAS)	IVW	0.033	0.003	0.001	1.003	1.0002–1.005
		WM	0.044	0.003	0.002	1.003	1.00006–1.007

SNPs: single nucleotide polymorphisms. MR: Mendelian randomization. IVW: inverse variance weighted. WM: weighted median. SE: standard error. b: standardized coefficient.

Under the condition of excluding genetic variations that exhibit a high association with established confounding factors (r^2^>0.8), Cochran’s Q test (IVW: Q_p=0.390) indicated an absence of heterogeneity in the causal analysis outcomes between smoking and RAS. The reliability of the results is further substantiated by the MR-Egger intercept (intercept= -0.0002, SE=0.0005, p=0.718). Our analysis did not reveal a significant association between the IV effect and the direct effect, thereby supporting the validity of the InSIDE assumption in our study. The MR-PRESSO multivariate pleiotropy tests yielded a global-test p=0.479, with an outlier-corrected output of NA (not applicable), indicating that no outliers were detected. Table 2 provides the results of sensitivity analyses of smoking and RAS. These findings suggest that there is no significant effect for RAS in the absence of smoking-related SNPs, and that SNPs do not exert their influence on outcomes through pathways independent of smoking exposure.

**Table 2:** Results from the sensitivity analysis concerning horizontal pleiotropy and heterogeneity tests in the MR study on smoking and recurrent aphthous stomatitis

*Exposure* *phenotype*	*Outcome* *phenotype*	*Horizontal pleiotropy*					*Heterogeneity*		
		*MR-Egger*			*MR-PRESSO*		*IVW*		
		*Intercept*	*SE*	*p*	*Global-test p*	*Outlier* *corrected*	*Q*	*Q_df-*	*Q_p*
Smoking	Recurrent aphthous stomatitis (RAS)	-0.0002	0.0005	0.718	0.479	NA	23.203	22	0.390

MR: Mendelian randomization. IVW: inverse variance weighted. SE: standard error. df: degrees of freedom. Q: Cochran’s Q. NA: not applicable.

Table 2 also provides the results of sensitivity analyses to test for horizontal pleiotropy and heterogeneity in the MR model. It includes results from the MR-Egger intercept, standard error, p-value, MR-PRESSO global-test p-value, outlier correction status, and IVW heterogeneity test (Q, degrees of freedom, and p-value). This analysis ensures the robustness of the MR findings by assessing potential biases and heterogeneity in the genetic instruments.

Furthermore, in the funnel plot demonstrates the heterogeneity of the effects of IVs. The leave-one-out analysis plot demonstrates that the exclusion of any individual SNP did not significantly affect the overall results (as shown in Supplementary file Figures 1 and 2, which include the funnel plot and the leave-one-out plot for sensitivity visualization).

## DISCUSSION

Through MR analysis, our study identified a potential causal relationship between smoking and the risk of developing RAS. The IVW method and the WM method yielded consistent ORs. Notably, both the OR value and the lower bound of the 95% CI were close to 1, suggesting that the increased risk of RAS associated with smoking exposure may be minimal. From a biological perspective, this implies that the effect of smoking on the incidence of RAS is relatively limited. Nevertheless, the statistical significance of the findings suggests that the observed positive correlation between smoking and RAS, though weak, is unlikely to be attributable to random error. Consequently, smoking may indeed contribute to the development of RAS. These findings underscore the importance of even minor increases in risk, particularly within the public health sector, where smoking remains a prevalent and modifiable risk factor. This is especially pertinent when considering the cumulative effects across large populations or over extended time periods, which may lead to substantial health impacts. The consistency observed across MR methods strengthens the reliability of the association and diminishes the likelihood of bias from pleiotropy or confounding factors, common challenges in observational studies. The findings produced have been corroborated through sensitivity analysis techniques, including assessments of heterogeneity and pleiotropy, indicating a high degree of accuracy and reliability. While the MR method theoretically mitigates the influence of confounders, we also employed the LDlink tool to minimize genetic variation associated with potential confounders. Nonetheless, in practice, certain latent genetic or environmental factors, as well as the heterogeneity in the etiology of RAS, may still impact the results. These factors may partially account for the observed weak positive association between smoking and RAS. Future research should aim to substantiate these initial findings and investigate the definitive role of smoking as a risk factor in the development of RAS.

Several observational studies suggest that smoking may protect against RAS. Mohamed et al.^22^ found that smokers had a lower prevalence of RAS, possibly due to nicotine’s effects on the immune system and oral mucosa keratinization. These findings should be viewed with caution due to possible confounding factors and the retrospective study design. The negative impact of smoking on systemic and oral health far outweighs any potential benefits. Our MR approach, aimed at reducing confounding, found no strong evidence of smoking having a protective effect on RAS. This highlights the complexity of the relationship and underscores the necessity for further research with robust designs to confirm these results and clarify the underlying mechanisms.

Smoking is associated with oxidative stress responses and is characterized by an elevated production of reactive oxygen species (ROS)^23,24^. Under normal physiological conditions, the oral mucosa modulates ROS production within physiological limits, through antioxidant systems, thereby preventing detrimental effects on tissues and organs^25^. In oral mucosal tissue, cells exposed to cigarette smoke produce a substantial amount of ROS, disrupting the equilibrium of the antioxidant system and inducing a state of oxidative stress in the oral mucosa. This imbalance favors oxidative mechanisms, allowing these highly reactive molecules to cause tissue damage through multiple pathways, including DNA damage, lipid peroxidation (LPO), and protein oxidation, ultimately contributing to the development of RAS^26^. Malondialdehyde (MDA), total oxidant status (TOS), and oxidative stress index (OSI) – calculated as the ratio of TOS to total antioxidant status (TAS) – serve as biomarkers of oxidative stress in RAS^27^. Empirical studies have demonstrated that serum levels of MDA, TOS, and OSI are significantly elevated in RAS patients compared to healthy controls^28^, suggesting a strong association between the pathogenesis of RAS and oxidative stress mechanisms. Furthermore, erythrocytes are critical for oxygen transport, and enzymes such as glutathione peroxidase (GPx), and superoxide dismutase (SOD) serve as primary antioxidants within these cells. These enzymes play an essential role in the oxidative stress defence mechanisms in the oral cavity and are associated with RAS^27^.

Research indicates that smokers demonstrate morphological abnormalities in red blood cells compared to non-smokers, including a reduction in red blood cell distribution width^29^. Furthermore, the activities of SOD and GPx in red blood cells, along with serum TAS, are markedly diminished in patients with RAS^28^. These findings suggest that smoking as a lifestyle factor can induce oxidative stress in the oral mucosa and compromise the mucosal epithelium’s defence mechanisms against oxidative stress.

Smoking is also a prevalent factor influencing oral microbiology, as cigarette smoke introduces numerous toxic substances that directly interact with oral microorganisms. These toxins can perturb the oral microbiome through mechanisms similar to those of antibiotics, hypoxia, or other potential pathways^30^. Wu et al.^31^ found that smokers’ oral microbiomes contained significantly fewer proteobacteria than those of non-smokers. Inferred metagenomic analysis suggests that this phenomenon may be attributed to the impact of smoking on oxygen availability in the oral cavity and the subsequent degradation of heterologous microorganisms^31^. Huang et al.^32^ identified variations in both α and β diversity of oral microbiota among smokers. Concurrently, other researchers have observed that the population of salivary streptococci may fluctuate due to perturbations in the oral environment, with a decrease in its abundance being associated with an increase in RAS incidence^33^. The oral microbial diversity in patients with RAS is diminished, and this dysbiosis is associated with metabolic disturbances involving metabolites such as amino acids, lipids, nucleotides, and caffeine. Metabolites, as critical constituents of the oral microbiome, have a direct impact on the survival and proliferation of oral microorganisms. The interactions between microorganisms and metabolites are markedly intensified, suggesting a significant association between oral microbiome dysbiosis, metabolic disorders, and the onset and progression of RAS^34^. In a study by Stehlikova et al.^35^, microbial dysbiosis was linked to the occurrence of RAS. The research demonstrated notable disparities in both α and β diversity of microbial communities when comparing RAS patients to healthy control subjects, based on the analysis of oral microbial samples. Notably, they observed a significant reduction in the abundance of streptococcus, a key constituent of the oral health microbiome^35^. In summary, smoking may increase the risk of RAS by inducing changes in the composition of the oral microbiome.

It is noteworthy that the oxidative stress response that smoking induces has been observed to activate specific proteases within the oral mucosal epithelial cells. Among these are proteases recognized for their cell-protective roles, possessing anti-inflammatory and antioxidant properties. These proteases are implicated in the detoxification of ROS, enhancement of cellular antioxidant capacity, and facilitation of anti-inflammatory processes. The principal mediator of this process is the transcription factor nuclear factor erythroid 2-related factor 2 (Nrf 2), which is expressed ubiquitously across various organisms^36^. In the quiescent state of cells, nuclear levels of Nrf 2 are maintained at a low concentration. Nonetheless, upon exposure of epithelial cells to oxidants generated by smoking, Nrf 2 is upregulated via phosphorylation and other biochemical pathways. This upregulation induces the expression of a suite of antioxidant enzyme genes, including GPx, which plays a vital role in mitigating the deleterious effects of ROS generated in the oral cavity. Simultaneously, the activation of Nrf 2 can enhance carbon monoxide production, upregulate SOD expression, induce the expression of mitochondrial antioxidant genes in oral mucosal epithelial cells, and modulate mitochondrial structure and function. These actions help mitigate oxidative stress-induced damage. It can be inferred that the Nrf 2 signaling pathway and its associated mechanisms exhibit significant potential for application in the treatment and prevention of RAS.

### Strengths and limitations

A key strength of our study lies in its use of publicly available GWAS databases characterized by large sample sizes. We utilized SNPs that have been meticulously selected according to stringent criteria to serve as exogenous proxies for smoking exposure. This methodological approach, augmented by MR analysis and a series of sensitivity analyses, substantiates the causal connection between smoking and RAS. By mitigating the effects of confounding variables and reverse causation, our study enhances the precision of causal inference concerning the smoking-RAS relationship, thereby addressing the limitations inherent in prior observational research. Moreover, the ongoing updates to the GWAS database contribute to the progressive nature of our research findings. Nevertheless, our study is not without its limitations. Smoking exposure was defined exclusively by the binary criterion of whether an individual smokes, without accounting for variables such as duration, intensity, or frequency of smoking. This simplified methodology may constrain our ability to comprehensively assess the impact of smoking on the risk of developing RAS. Future research should incorporate more nuanced measures of smoking exposure, such as secondhand smoking, smoking intensity, or duration, to enhance the understanding of the relationship between smoking and the incidence of RAS. We recognize that RAS has varied causes, influenced by factors like recurrence, immune status, or comorbidities, which could affect the link between smoking and RAS. Our study examined genetic predisposition to smoking without considering other factors like recurrence or immune status.

Future research should investigate how these varied factors interact with smoking to affect RAS development. Moreover, the data sets used in our research are from particular areas and demographic groups, which might restrict the wider relevance of the conclusions. Genetic variation tools associated with smoking are often derived from specific populations and may not comprehensively capture the diversity of smoking behaviors across different demographic groups or cultural contexts. Future research should aim to incorporate more datasets to enhance the generalizability and strength of the findings. Furthermore, most GWAS databases do not provide differentiation by age groups, and the spectrum of disease severity is broad, which constrains the potential for stratified analysis. Additionally, although the application of a p-value threshold of p<5×10^-6^ as a screening criterion increased the number of SNPs analyzed, it also introduced a potential bias in the data.

## CONCLUSIONS

We have provided initial evidence indicating a possible causal link between smoking and RAS. Our findings highlight the necessity for additional investigation into the role of smoking in the etiology of RAS. Subsequent research should expand upon these results to confirm the causal relationship and explore other contributing factors to the incidence of RAS cases. This will facilitate the development of targeted public health strategies and enhance management approaches for individuals at risk of RAS.

## Supplementary Material



## Data Availability

The GWAS data used in this study are publicly available from the UKB and FinnGen research teams. Additional data supporting this research can be found in the Supplementary file.
