# Supplementary material for: The relationship between smoking and recurrent aphthous stomatitis: A Mendelian randomization study
Source: Tob Induc Dis. 2025 Jan 15;23:10.18332/tid/199253. doi: 10.18332/tid/199253 (PMC11734161; doi:10.18332/tid/199253)
Supplement: Supplementary file 1 [file TID-23-02-s1.pdf]

Supplementary Materials

Supplementary Table 1-3

Supplementary Table 1.Summary data from all GWAS used in current study.

Phenotype	Sample ize	Ethnic	Cohort source	Number of SNPs
Smoking	166,614	EUR	Finn	21,288,424
Recurrent aphthous stomatitis	498,697	EUR	British	13,791,467

GWAS, Genome-Wide Association Study;EUR, European;Finn, obtained from the Finnish database;British,obtained from the UK Biobank;SNPs,Single Nucleotide Polymorphisms

This table Summarizes the GWAS data sources used to investigate the relationship between smoking and RAS. Provides an overview of the foundational data. Contains information on each phenotype's GWAS, including sample size, ethnicity of the cohort, source of the data, and the number of SNPs used for each phenotype.

Supplementary Table 2. Detailed information about SNPs's LDlink in this study.

Num.	SNPs	GWAS Trait	PMID	rs Number	Position(GRCh37)	r^2	P-value
1	rs7556895	Age-related cognitive decline (visuospatial skill) (slope of z-scores)	30954325	rs77993098	chr2:35063984	0.828	4x10^-6
2	rs13394375	Heart failure	20445134	rs13418717	chr2:127662897	0.930	3x10^-6
3	rs10005036	NA					
4	rs11096778	Educational attainment	35361970	rs55900829	chr4:35514712	0.912	1x10^-25
		Educational attainment (years of education)	30038396	rs317050	chr4:35446302	0.908	3x10^-8
		Highest math class taken (MTAG)	30038396	rs317050	chr4:35446302	0.908	2x10^-12
		Self-reported math ability (MTAG)	30038396	rs317056	chr4:35431837	0.823	5x10^-9
5	rs56246836	NA					
6	rs6849973	NA					
7	rs115192	NA					
8	rs2517549	NA					
9	rs118075160	NA					
10	rs10256402	NA					
11	rs6478058	NA					
12	rs117210485	NA					

Num.	SNPs	GWAS Trait	PMID	rs Number	Position(GRCh37)	r²	P-value
13	rs10762774	NA					
14	rs2127307	NA					
15	rs67411117	NA					
16	rs117104143	Disease-related mortality after unrelated donor allogeneic hematopoietic cell transplantation (donor effect)	34746714	rs79076914	chr14:60559239	0.926	3x10^-8
17	rs12890188	NA					
18	rs72740955	Post bronchodilator FEV1/FVC ratio or FEV1	26634245	rs2036527	chr15:78851615	0.999	9x10^-15
				rs55853698	chr15:78857939	0.987	7x10^-14
				rs55781567	chr15:78857986	0.992	3x10^-6
				rs58365910	chr15:78849034	0.987	7x10^-15
				rs72738786	chr15:78828086	0.979	8x10^-14
				rs8031948	chr15:78816057	0.946	3x10^-15
				rs931794	chr15:78826180	0.946	2x10^-11
				rs7180002	chr15:78873993	0.941	1x10^-15
				rs17486195	chr15:78865197	0.941	2x10^-15
				rs17486278	chr15:78867482	0.941	3x10^-18

Num.	SNPs	GWAS Trait	PMID	rs Number	Position(GRCh37)	r²	P-value
				rs140330585	chr15:78866445	0.941	1x10^-15
				rs951266	chr15:78878541	0.941	1x10^-15
				rs7172118	chr15:78862453	0.941	2x10^-15
				rs34684276	chr15:78813155	0.937	2x10^-11
				rs56390833	chr15:78877381	0.937	2x10^-15
				rs72740964	chr15:78868636	0.933	8x10^-15
				rs11633958	chr15:78862064	0.929	3x10^-14
				rs10519203	chr15:78814046	0.929	6x10^-12
	rs72740955	Post bronchodilator FEV1/FVC ratio or FEV1		rs55676755	chr15:78898932	0.921	2x10^-17
				rs8034191	chr15:78806023	0.921	3x10^-9
				rs1051730	chr15:78894339	0.921	2x10^-15
				rs138544659	chr15:78900701	0.917	3x10^-16
				rs8192482	chr15:78886198	0.917	6x10^-15
				rs147144681	chr15:78900908	0.917	6x10^-17
				rs16969968	chr15:78882925	0.917	6x10^-15
				rs4887067	chr15:78886947	0.917	5x10^-15

Num.	SNPs	GWAS Trait	PMID	rs Number	Position(GRCh37)	r²	P-value
		Post bronchodilator FEV1/FVC ratio or FEV1		rs146009840	chr15:78906177	0.913	2x10^-15
				rs1317286	chr15:78896129	0.909	3x10^-14
				rs56077333	chr15:78899003	0.909	6x10^-6
				rs4243084	chr15:78911672	0.897	8x10^-14
				rs9788721	chr15:78802869	0.878	2x10^-14
				rs72743158	chr15:78926445	0.811	3x10^-13
	rs72740955	Cotinine and 3'-hydroxycotinine levels in current smokers	32157176	rs2036527	chr15:78851615	0.999	3x10^-28
		Pulmonary function	21946350	rs2036527	chr15:78851615	0.999	7x10^-7
		Lung cancer	39366959	rs2036527	chr15:78851615	0.999	8x10^-179
			27393504	rs55781567	chr15:78857986	0.992	1x10^-9
			28604730	rs55781567	chr15:78857986	0.992	3x10^-103
			35915169	rs55781567	chr15:78857986	0.992	6x10^-106
			39366959	rs17486278	chr15:78867482	0.941	2x10^-17
			18780872	rs8034191	chr15:78806023	0.921	1x10^-8
			18978790	rs1051730	chr15:78894339	0.921	1x10^-15
			32887889	rs8042849	chr15:78817929	0.883	8x10^-10

Num.	SNPs	GWAS Trait	PMID	rs Number	Position(GRCh37)	r^2	P-value
	rs72740955	Lung cancer in ever smokers	28604730	rs55781567	chr15:78857986	0.992	2x10^-78
		Lung adenocarcinoma	32889700	rs55781567	chr15:78857986	0.992	9x10^-26
			28604730	rs55781567	chr15:78857986	0.992	3x10^-48
			35915169	rs55781567	chr15:78857986	0.992	2x10^-50
			19836008	rs1051730	chr15:78894339	0.921	2x10^-51
		Lung adenocarcinoma (conditioned on cigarettes per day)	39366959	rs2036527	chr15:78851615	0.999	3x10^-82
		Non-small cell lung cancer	31326317	rs55781567	chr15:78857986	0.992	8x10^-44
		Squamous cell lung carcinoma	32889700	rs55781567	chr15:78857986	0.992	2x10^-23
			35915169	rs55781567	chr15:78857986	0.992	2x10^-33
		Squamous cell lung carcinoma (conditioned on cigarettes per day)	39366959	rs55781567	chr15:78857986	0.992	2x10^-74
		Urate levels	31578528	rs55781567	chr15:78857986	0.992	1x10^-8
		Aerodigestive squamous cell cancer (pleiotropy)	33667223	rs55781567	chr15:78857986	0.992	2x10^-29
		Small cell lung carcinoma	28604730	rs55853698	chr15:78857939	0.987	5x10^-21
			35915169	rs16969968	chr15:78882925	0.917	1x10^-23
		Abdominal aortic aneurysm	36777996	rs58365910	chr15:78849034	0.987	1x10^-13
			37845353	rs17486278	chr15:78867482	0.941	2x10^-35

Num.	SNPs	GWAS Trait	PMID	rs Number	Position(GRCh37)	r²	P-value
	rs72740955	Airflow obstruction	32981348	rs55958997	chr15:78915872	0.807	9x10^-14
			22837378	rs8031948	chr15:78816057	0.946	3x10^-9
			22837378	rs17486278	chr15:78867482	0.941	2x10^-7
		Parental longevity (father's age at death)	31484785	rs931794	chr15:78826180	0.946	3x10^-9
			29227965	rs951266	chr15:78878541	0.941	6x10^-14
		Parental longevity (both parents in top 10%)	29227965	rs951266	chr15:78878541	0.941	1x10^-9
		Parental longevity (combined parental age at death)	29227965	rs951266	chr15:78878541	0.941	1x10^-11
		Parental longevity (combined parental attained age, Martingale residuals)	29227965	rs1317286	chr15:78896129	0.909	1x10^-26
		Parental lifespan	29030599	rs8042849	chr15:78817929	0.883	4x10^-14
		Chronic obstructive pulmonary disease liability (machine learning-based score)	37069358	rs931794	chr15:78826180	0.946	3x10^-8
		Chronic obstructive pulmonary disease	28166215	rs17486278	chr15:78867482	0.941	2x10^-28
			35308900	rs72740964	chr15:78868636	0.933	2x10^-16
			30804561	rs55676755	chr15:78898932	0.921	3x10^-26
			19300482	rs8034191	chr15:78806023	0.921	1x10^-10

Num.	SNPs	GWAS Trait	PMID	rs Number	Position(GRCh37)	r^2	P-value
			33909500	rs16969968	chr15:78882925	0.917	5x10^-25
			34594039	rs9788721	chr15:78802869	0.878	4x10^-38
		Peripheral artery disease in non diabetes	34601942	rs931794	chr15:78826180	0.946	4x10^-10
			34601942	rs1051730	chr15:78894339	0.921	9x10^-9
		Pneumothorax	34594039	rs931794	chr15:78826180	0.946	4x10^-8
		Pulmonary artery enlargement and chronic obstructive pulmonary disease	25101718	rs17486278	chr15:78867482	0.941	7x10^-10
	rs72740955	Local histogram emphysema pattern	25006744	rs17486278	chr15:78867482	0.941	8x10^-13
			25006744	rs11852372	chr15:78801394	0.872	2x10^-10
		Diffusing capacity of carbon monoxide	30694715	rs17486278	chr15:78867482	0.941	9x10^-10
			30694715	rs112878080	chr15:78900647	0.917	3x10^-10
		Mortality	27029810	rs10519203	chr15:78814046	0.929	2x10^-17
		Fibrinogen levels	25551457	rs10519203	chr15:78814046	0.929	6x10^-8
		Intracranial aneurysm	33199917	rs10519203	chr15:78814046	0.929	1x10^-9
		Emphysema imaging phenotypes	26030696	rs55676755	chr15:78898932	0.921	2x10^-9
			26030696	rs9788721	chr15:78802869	0.878	1x10^-6

Num.	SNPs	GWAS Trait	PMID	rs Number	Position(GRCh37)	r^2	P-value
	rs72740955	Intracranial aneurysm	33199917	rs8034191	chr15:78806023	0.921	3x10^-8
		Poultry consumption	32193382	rs1051730	chr15:78894339	0.921	5x10^-9
		Cryptic phenotype that captures alpha-1-antitrypsin deficiency severity	35760791	rs16969968	chr15:78882925	0.917	3x10^-12
		Menarche (age at onset)	30595370	rs4243084	chr15:78911672	0.897	1x10^-7
		Local histogram emphysema pattern	25006744	rs9788721	chr15:78802869	0.878	2x10^-13
		Exhaled carbon monoxide levels	25072098	rs55958997	chr15:78915872	0.807	2x10^-9
19	rs73575193	Body mass index	36581621	rs4985407	chr16:70285901	0.914	7x10^-26
			31669095	rs2070203	chr16:70303580	0.896	9x10^-19
			37280435	rs775208	chr16:70315911	0.891	9x10^-20
			39134668	rs9939726	chr16:70440559	0.858	3x10^-17
		Body mass index (MTAG)	36376304	rs775208	chr16:70315911	0.891	3x10^-19
		Weight	34594039	rs775208	chr16:70315911	0.891	2x10^-10
		Body mass index or knee osteoarthritis (pleiotropy)	36889626	rs775208	chr16:70315911	0.891	2x10^-22
		Triglyceride levels (MTAG)	36376304	rs936994	chr16:70442408	0.862	3x10^-8
		Youthful appearance (self-reported)	32339537	rs9924898	chr16:70241566	0.851	5x10^-8
20	rs58356259	Morningness	30804565	rs58356259	chr17:8040151	0.999	2x10^-9

Num.	SNPs	GWAS Trait	PMID	rs Number	Position(GRCh37)	r^2	P-value
		High light scatter reticulocyte percentage of red cells	32888494	rs58356259	chr17:8040151	0.999	2x10^-11
21	rs72927099	NA					
22	rs4645887	NA					
23	rs11700623	NA					

SNPs,Single Nucleotide Polymorphism; GWAS,genome-wide association study; PMID,publication references; r^2 ,degree of explanation.

The table provides detailed confounding factor information for the SNPs analyzed in this study, including their GWAS traits, rs numbers, genomic locations, linkage disequilibrium (r^2), and p-values. It retains all confounding factor information for SNPs with linkage disequilibrium(LD) r^2>0.8. This helps identify genetic markers that are highly correlated with potential confounding factors.

Supplementary Table 3. Details of the SNPs in this study.

smoking.exposure	SNPs	other_allele	effect_allele	pval.exposure	beta.exposure	se.exposure	eaf.exposure	maf.exposure	R^2	F	pval.[illegible]
1	rs7556895	A	C	1.83E-06	0.123173	0.0258175	0.251486	0.748514	0.005711823	957.125259	0.34[illegible]
2	rs13394375	C	T	3.06E-06	0.285194	0.0611117	0.0302162	0.9697838	0.004766785	798.0074609	0.87[illegible]
3	rs10005036	G	T	7.83E-08	-0.161916	0.0301469	0.186927	0.813073	0.007969134	1338.419318	0.76[illegible]
4	rs11096778	T	C	1.99E-06	0.109032	0.0229329	0.526944	0.473056	0.005926728	993.3512723	0.87[illegible]
5	rs56246836	T	C	8.35E-07	0.124278	0.0252233	0.271295	0.728705	0.006106777	1023.713886	0.68[illegible]
6	rs6849973	C	G	3.66E-06	-0.15791	0.0341085	0.138407	0.861593	0.005947158	996.7959995	0.3[illegible]
7	rs115192	G	A	2.60E-06	0.12202	0.02596	0.248223	0.751777	0.005556779	930.9993499	0.12[illegible]
8	rs2517549	C	A	1.41E-08	0.143582	0.0253136	0.270804	0.729196	0.008141967	1367.685076	0.27[illegible]
9	rs118075160	A	G	3.71E-07	0.925297	0.182033	0.00208064	0.99791936	0.003555369	594.4807576	0.22[illegible]
10	rs10256402	T	C	4.97E-06	-0.191613	0.041965	0.0879466	0.9120534	0.005890054	987.1681079	0.13[illegible]
11	rs6478058	T	C	3.03E-07	0.170521	0.0332949	0.123539	0.876461	0.006296836	1055.776571	0.85[illegible]
12	rs117210485	G	A	4.03E-06	-0.174279	0.0378054	0.113925	0.886075	0.006132106	1027.986121	0.94[illegible]
13	rs10762774	G	A	2.40E-06	0.108529	0.023012	0.422013	0.577987	0.005745998	962.885016	0.08[illegible]
14	rs2127307	C	A	3.77E-07	-0.143769	0.0283003	0.221104	0.778896	0.007119288	1194.663999	0.81[illegible]

smoking.exposure	SNPs	other_allele	effect_allele	pval.exposure	beta.exposure	se.exposure	eaf.exposure	maf.exposure	R^2	F	pval.o[illegible]
15	rs67411117	A	T	2.83E-06	-0.148282	0.0316642	0.166379	0.833621	0.006099216	1022.43867	0.34[illegible]
16	rs117104143	A	C	4.41E-06	-0.352318	0.0767419	0.0275068	0.9724932	0.00664089	1113.848954	0.42[illegible]
17	rs12890188	A	G	8.88E-07	-0.112393	0.0228672	0.486097	0.513903	0.00631121	1058.201819	0.39[illegible]
18	rs72740955	C	T	1.29E-12	0.168848	0.0237957	0.335734	0.664266	0.012716254	2145.96916	0.05[illegible]
19	rs58356259	C	T	3.80E-06	0.105948	0.0229235	0.452781	0.547219	0.005562434	931.9522038	0.77[illegible]
20	rs73575193	C	T	1.78E-06	0.115995	0.0242802	0.653031	0.346969	0.006097236	1022.104702	0.76[illegible]
21	rs72927099	T	C	2.40E-06	0.1472	0.0312109	0.15007	0.84993	0.005527422	926.0535963	0.03[illegible]
22	rs4645887	T	A	1.19E-06	-0.124053	0.0255402	0.297999	0.702001	0.006438683	1079.713867	0.33[illegible]
23	rs11700623	A	G	3.55E-08	-0.137642	0.0249713	0.316686	0.683314	0.008199382	1377.409364	0.04[illegible]

SNPs,Single Nucleotide Polymorphism; beta,the effect size of the estimated effect allele on the exposure; se,standard error; eaf,the effect allele frequency; maf,the minor allele frequency; R2,degree of explanation; F,statistical magnitude.

This table lists the specific SNPs used in the analysis of the relationship between smoking and RAS. Each row represents a SNPs associated with smoking exposure. The columns include the SNPs identifier, other allele, effect allele, p-value of exposure, beta coefficient for exposure, se, eaf, maf, R^2 , F-statistic, outcome p-value, and harmonization status (retain or eliminate).Provides genetic variant details essential for understanding the Mendelian randomization analysis setup. One SNPs (rs10820003) was excluded due to its palindromic structure and moderate frequency of the effect allele.

Supplementary Figure 1. Funnel Plot for assessing the symmetry and potential publication bias in Two-Sample MR analysis of smoking and RAS.

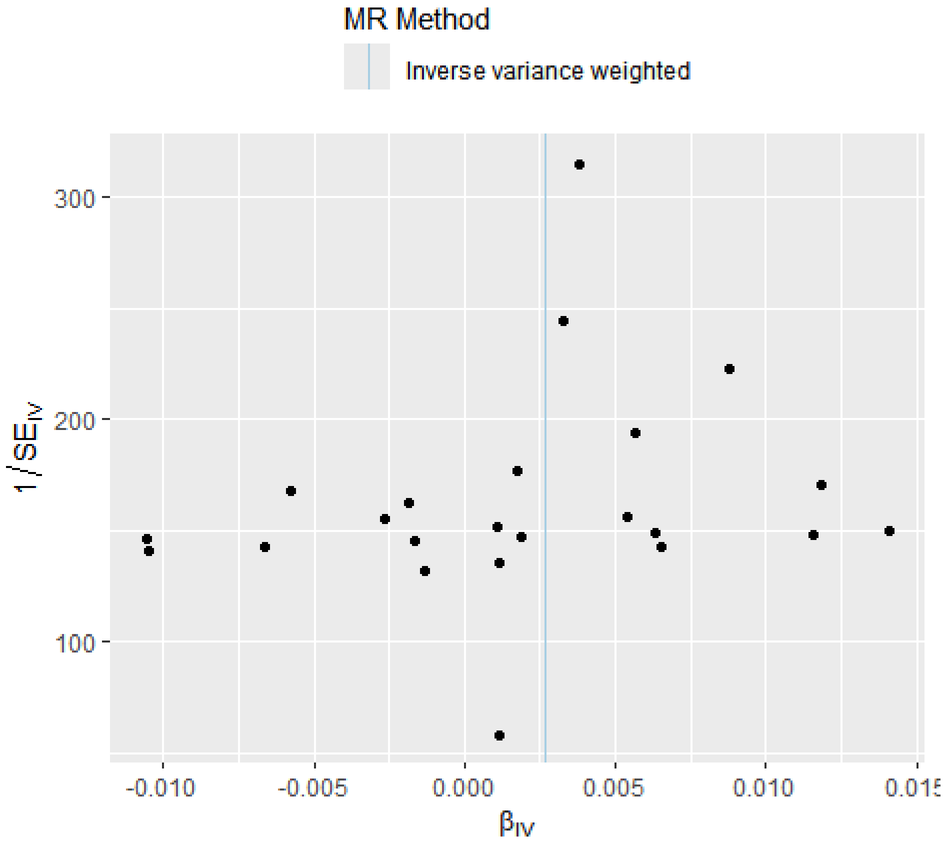

The funnel plot of single-SNPs effect estimates and corresponding inverse standard errors. This funnel plot displays results from the IVW method in MR. The x-axis shows the effect estimate, while the y-axis represents the inverse of the standard error. Each dot corresponds to an individual SNPs, reflecting its effect estimate and precision.

Supplementary Figure 2. Leave-one-out sensitivity analysis of two-sample MR for assessing the robustness of the association between smoking and RAS

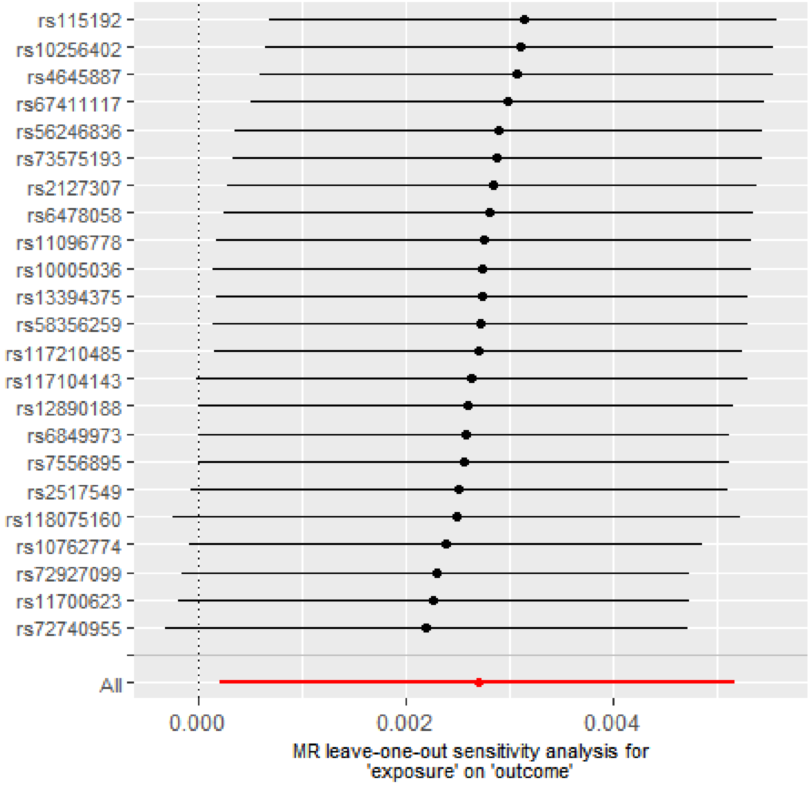

Excludes the possibility of causation due to the main effect of SNPs. The x-axis represents the effect estimate of the exposure on the outcome, and each horizontal line corresponds to an individual SNP. The red line represents the overall effect estimate when all SNPs are included. SNPs: single nucleotide polymorphisms. RAS: recurrent aphthous stomatitis. MR: Mendelian randomization.

©2025 Hu Y. et al.
